# Monumental megalithic burial and rock art tell a new story about the Levant Intermediate Bronze “Dark Ages”

**DOI:** 10.1371/journal.pone.0172969

**Published:** 2017-03-02

**Authors:** Gonen Sharon, Alon Barash, Davida Eisenberg-Degen, Leore Grosman, Maya Oron, Uri Berger

**Affiliations:** 1 Prehistory Laboratory East Campus, Tel Hai College, Tel Hai, Upper Galilee, Israel; 2 Faculty of Medicine in the Galilee, Bar Ilan University, Safed, Israel; 3 Southern District, Israel Antiquities Authority, Jerusalem, Israel; 4 Computerized Archeology Laboratory, Institute of Archaeology, The Hebrew University of Jerusalem, Jerusalem, Israel; 5 Department of Prehistory, Israel Antiquities Authority, Jerusalem, Israel; 6 Northern District, Israel Antiquities Authority, Jerusalem, Israel; Seoul National University College of Medicine, REPUBLIC OF KOREA

## Abstract

The Intermediate Bronze Age (IB) in the Southern Levant (ca. 2350–2000 BCE) is known as the “Dark Ages,” following the collapse of Early Bronze urban society and predating the establishment of the Middle Bronze cities. The absence of significant settlements and monumental building has led to the reconstruction of IB social organization as that of nomadic, tribal society inhabiting rural villages with no central governmental system. Excavation in the Shamir Dolmen Field (comprising over 400 dolmens) on the western foothills of the Golan Heights was carried out following the discovery of rock art engravings on the ceiling of the central chamber inside one of the largest dolmens ever recorded in the Levant. Excavation of this multi-chambered dolmen, covered by a basalt capstone weighing some 50 tons, revealed a secondary multi-burial (of both adults and children) rarely described in a dolmen context in the Golan. Engraved into the rock ceiling above the multi-burial is a panel of 14 forms composed of a vertical line and downturned arc motif. 3D-scanning by structured-light technology was used to sharpen the forms and revealed the technique employed to create them. Building of the Shamir dolmens required a tremendous amount of labor, architectural mastery, and complex socio-economic organization well beyond the capacity of small, rural nomadic groups. The monumental megalithic burial of the Shamir dolmens indicates a hierarchical, complex, non-urban governmental system. This new evidence supports a growing body of recent criticism stemming from new discoveries and approaches that calls for rethinking our views of the Levantine IB “Dark Ages.”

## Introduction

The Intermediate Bronze Age (IB) of the Southern Levant is known as the “Dark Ages.” The large cities of the Early Bronze Age, which were the region’s first urban settlements, collapsed and were abandoned. The complex socio-economic strategies, based on large-scale agriculture, industry, and trade, were replaced by what Dever [1] named “small-scale mixed agro-pastoralism.” This Dark Ages (also termed Early Bronze IV and Middle Bronze I) of pastoral nomadism lasted from ca. 2350 until 2000 BCE, when it was replaced by the urban renaissance of the Middle Bronze Age. The accepted interpretation of the archaeological record suggests that the socio-economic structure of the period declined into a primarily nomadic, tribal society inhabiting small rural villages. The economy is thought to have been based on herding. No evidence for strong central administration has been recovered. Almost no settlement of the period can be described as an urban center and, remarkably, there is no evidence recorded for monumental architecture [1–3].

Against this background of pastoral nomadism, the primary evidence left behind by Levantine IB communities is its vast and impressive burial grounds, scattered across the Southern Levant [4]. Among their multiple and varied burial praxes, the use of megalithic dolmens stands out as the most prominent, yet poorly understood, feature of this enigmatic period. Excavation in the Shamir Dolmen Field, located on the western foothills of the Golan Heights, was carried out following the 2012 discovery of engravings in one of the largest dolmens recorded in the Levant. This dolmen revealed evidence of a hierarchical, non-urban governmental system. This new evidence supports a growing body of recent criticism, stemming from new discoveries and approaches (e.g. [4, 5]), that calls for rethinking our views of the Levantine IB “Dark Ages.”

The traditional approach to understanding social complexity and level of governmental organization in archaeology is based primarily on the magnitude of settlements and the architectural achievements reflected in monumental structures. For example, the Natufian of the Levant built the earliest hamlets and were the world’s first settlers. However, the dramatic shift to a sedentary way of life is commonly assigned to the Neolithic mega-site, indicating a chiefdom-scale political organization (e.g. [6]). Similarly, the establishment of the first urban tells in the Levantine Early Bronze indicates a growing social and political organization. The implication, therefore, of the abandonment of settlements and an archaeological record void of large urban settlements and monumental public structures, is the collapse of a cultural system and a socio-political system of low order [7]. History, however, has numerous opposing examples. The largest empire of all time, the Mongolian Empire, was established by small groups of nomadic, tent dwellers. Other highly complex societies left their mark on the landscape not in the form of urban settlements but rather in the shape of ceremonial and religious monuments. The small chiefdoms of Easter Island established a complex and well-organized governmental system. Yet, they left behind not a single urban site (e.g. [7]). Evidence for their sophisticated culture and organization is found in the famous Moai statues and Ahu stone platforms. The Shamir dolmens tell a similar story about the “Dark Ages” of the Levantine IB.

### The Dolmen Phenomenon in the Levant

Megalithic stone structures were built by diverse cultural entities, during different periods, in places ranging from Ireland to Korea. Many attempts have been made to describe and classify this large variety of structures and compounds (for recent overview see [3]). Even when the discussion is limited to megalithic burial structures, they still vary in shape, size, rock type, stonemasonry, and architectural design. Beginning as early as the mid-nineteenth century, scholars have used terms such as dolmen, cist burial, cromlech, and triliton to describe megalithic burial structures (for overview of research history see [3,8,9]). In this study of the Levantine dolmens of the Shamir Dolmen Field we define a dolmen as a megalithic burial structure constructed from unhewn mega-stones with no cementation between them.

Dolmens are found in the Levant from Turkey to Egypt and as far east as the Arabian Desert [10,11]. Levantine dolmens were first observed by travelers and researchers in the early nineteenth century and it seems that almost all scholars and explorers of the Levant contributed to their study) e.g. [12–14]. In the Levant, large numbers of megalithic burial structures have been found, primarily in Syria, Jordan, and Israel, in defined areas known as dolmen fields. Interestingly, almost no dolmens have been found west of the Jordan Rift Valley. In the recently published high resolution survey of the Golan Heights [9,15], approximately 5200 structures defined as megalithic burials were identified, most of which are dolmens. The actual number of megalithic burials in the region is estimated to be much higher, as this number does not include large dolmen fields at the foothills of the Golan Heights (such as the Shamir Dolmen and the Kurazim Fields [16]). In comparison, the total number of dolmens recorded in the entire Western Caucasus is estimated to be 3000 [17]. The large number of Golan dolmens and their wide geographical distribution led Hartal [15] to suggest abandoning the term dolmen field, as it is impossible to define the border between different fields. He suggested viewing the Golan as a single, giant dolmen field.

### The Shamir Dolmen Field

**Fig 1:**
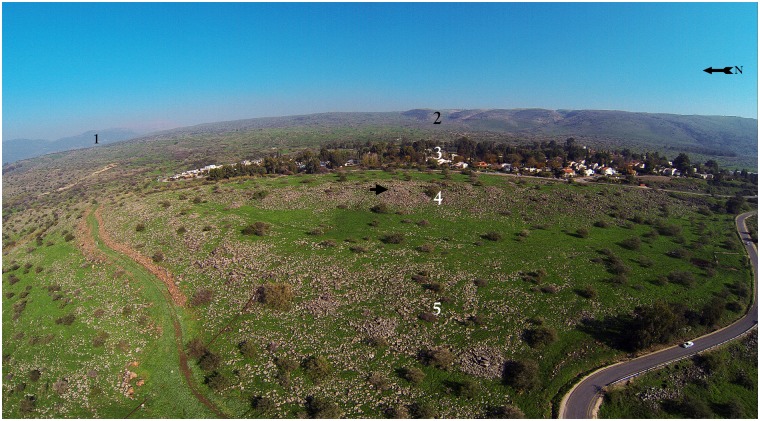
Aerial view of the Shamir Dolmen Field. (1) Mount Hermon; (2) The Golan Heights; (3) Kibbutz Shamir; (4) The upper basalt flow step; (5) The lower flow basalt step; note dolmens scattered on the steps.

The Shamir Dolmen Field surrounds Kibbutz Shamir, located on the lower western slopes of the Golan Heights in Northern Israel. The field covers a few square kilometers and was built on Middle Pleistocene Age basalt flows [18]. The flows formed into a series of large slab and giant boulder shaped steps slanting westward into the Hula Valley, some 150 meters below (Fig 1). These large slabs were the ideal raw material for dolmen construction. Many of the dolmens were built directly on the slopes, simplifying transport of the giant stones. Yet, numerous dolmens were also built on the plateaus between and above the slopes (Fig 1), meaning that the giant rocks were carried uphill a considerable distance to their final position.

**Fig 2:**
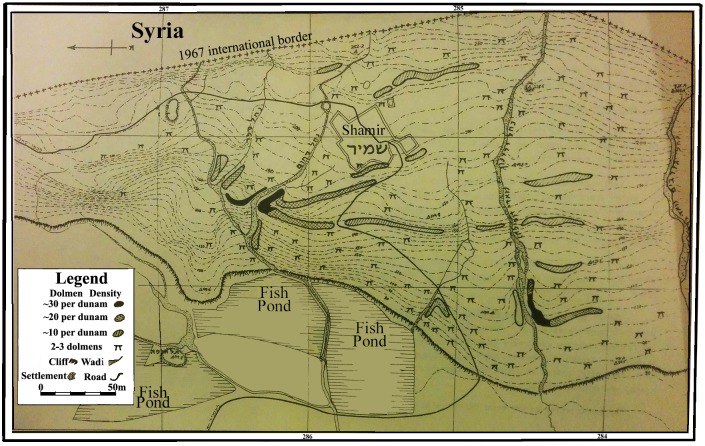
Map of Shamir Dolmen Field (after Kagan 1960 [19]).

Kagan, who in 1960 conducted the first survey and study of the Shamir Dolmen Field, counted approximately 400 megalithic structures overlooking the Hula Valley to the west [19] (Fig 2). Yet, apart from salvage excavations of seven dolmens that yielded only scattered finds [20–24], very little is known about these mysterious megalithic structures. Their chronology, construction technology, and even their actual usage for burial have been the subject of debate [3,8,20,25]. The finds from these dolmens include only a few ceramic sherds, which have been attributed to the IB [20–24]. Of special interest is a burial cave found in the Shamir Dolmen Field. Similar to the finds from the excavated dolmens, the ceramic assemblage from the cave was assigned to the ‘northern family’ of typical IB ceramics and the metal finds, including pins and an open bracelet with snake head ends, are well correlated with other IB assemblages in the region, including the Golan dolmens [24].

#### Dolmen classification

**Fig 3:**
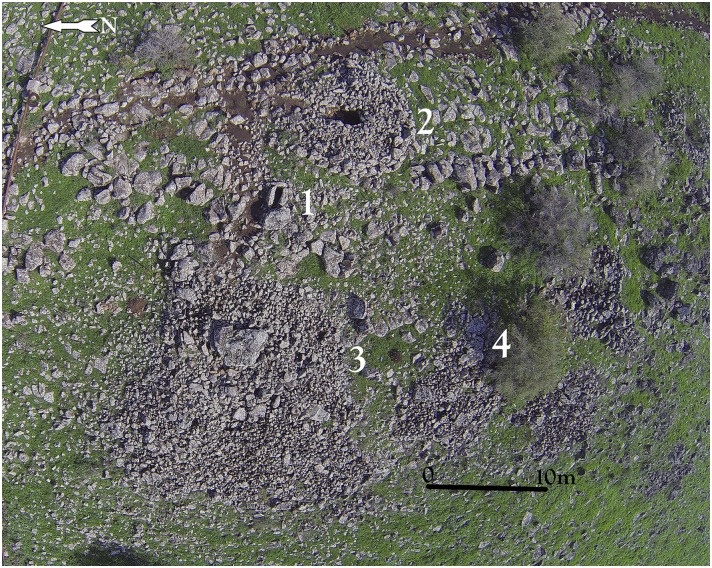
Aerial photo of the study area indicating the location of Dolmens 1–4.

**Fig 4:**
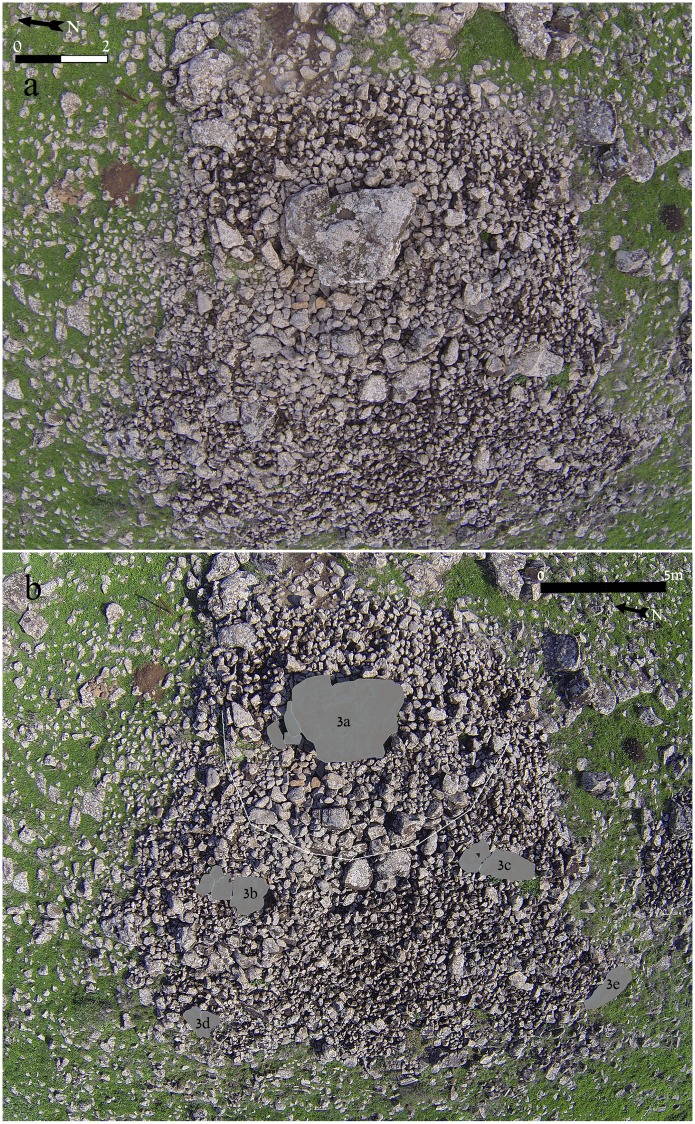
Dolmen 3. a. General view from above; scale 2 meters. b. Location of Dolmen 3 central chamber 3a and sub-chambers 3b to 3e; scale 5 meters.

Researchers have classified Levantine dolmens into various types [25,26,27]. The Shamir Dolmen Field includes most of these, at times scattered within a few meters of one another (Fig 3). The most distinct type of Shamir dolmen is a circular tumulus, featuring a well-built, surrounding wall constructed from massive stones that bounds a large number of smaller stones forming the tumulus. In the center of the tumulus is a large central chamber entered through a vestibule and covered with a giant capstone. Many of the massive capstones in the Shamir Dolmen Field rise out of their tumulus, most likely designed by the dolmen builders as a prominent landmark, visible even today (Fig 1). While this is the most prominent dolmen type of the Shamir Dolmen Field, other types are found as well. Six of the seven dolmens excavated previously at the Shamir Dolmen Field [20–23] belong to the smaller type (except for a single, very large dolmen excavated by Bahat [20]). Our study area, marking only c. 50 square meters, contained four different dolmen types next to each other (Fig 3). Hundreds of dolmens are scattered in the Shamir Dolmen Field, yet one dolmen stands out, even among the giants. The largest of the Shamir dolmens and, to our knowledge, one of the largest dolmens ever reported from the Levant, is Dolmen 3 (Fig 4).

## Results

### Architecture of Dolmen 3

The architecture of Dolmen 3 is that of the circular tumulus described above but is unique in its size. The Dolmen 3 tumulus, built around a central chamber (3a), is 20 meters in diameter (Fig 4). The total weight of the basalt stones used is estimated at ca. 400 tons. The giant tumulus of Dolmen 3 was built on a slope slanting steeply toward the west. The lower, western part of the tumulus partially collapsed, making it possible to identify two distinct construction stages (Fig 4). The first stage, marked by a circular line of giant boulders, can be observed at approximately half the diameter of the tumulus toward the west. At this line, two smaller sub-chambers (3b and 3c) were built into the corners of the tumulus. In the second stage, additional boulders and stones were piled to the west to enlarge the tumulus to its final dimensions and current state. As in stage one, two additional sub-chambers (3d and 3e) were constructed at the southwest and northwest corners of the tumulus (Fig 4b). It is unknown how much time, if any, transpired between the two construction stages. The four sub-chambers built into the Dolmen 3 tumulus are each medium-sized (c. 1 X 3 meters) and elongated, and covered by one to three massive basalt capstones.

**Fig 5:**
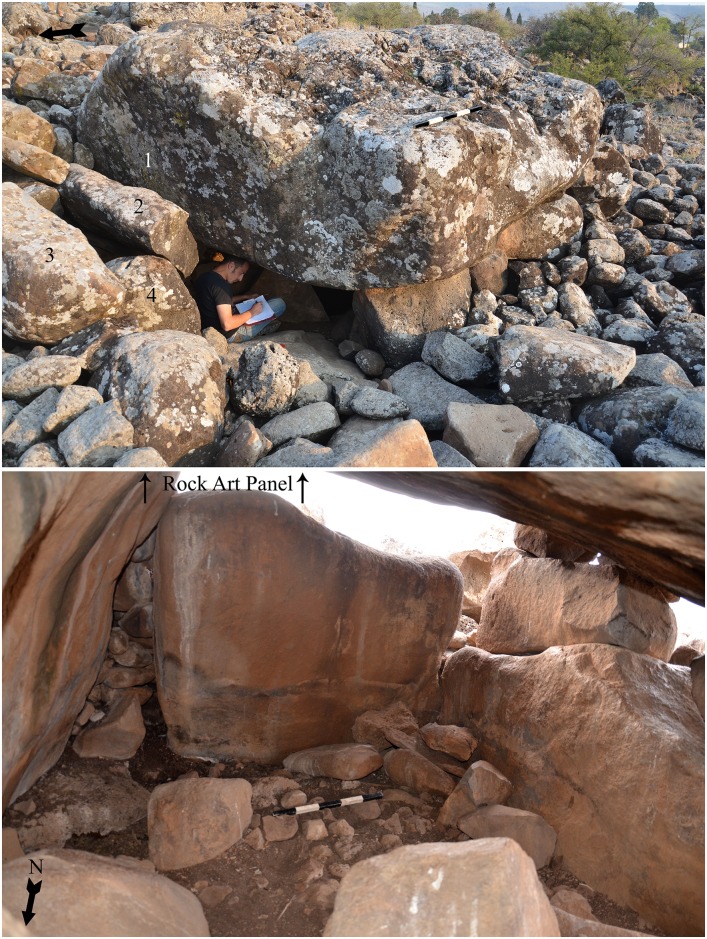
Dolmen 3a central chamber. a. Outside view looking southeast. 1. capstone; 2–3. corridor covering slabs; 4. corridor wall stone. b. Inner view of chamber 3a before excavation; location of rock art panel is indicated; scale 50cm.

**Fig 6:**
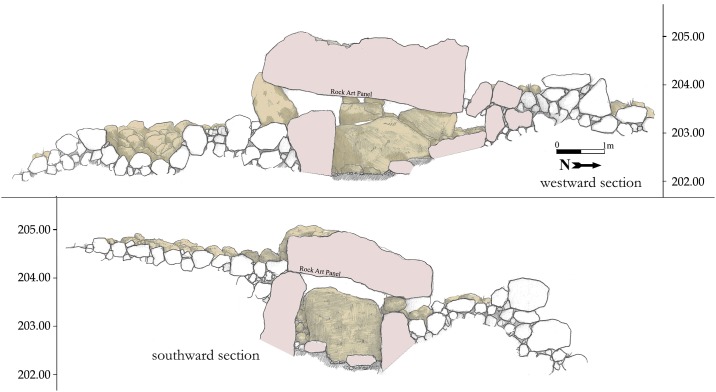
Westward and southward sections of Dolmen 3 central Chamber 3a.

In the upper part of the Dolmen 3 tumulus is the central chamber 3a. The chamber is rectangular, 3 meters long by 2 meters wide, and the ceiling is ca. 1.7 meters above the present day surface prior to excavation (Figs 5 and 6). To the north is a corridor entrance, roofed by elongated massive basalt slabs (Fig 5a) and blocked by stones. The present-day entrance to the dolmen is through a gap created by a fallen standing stone at the northwest corner of the chamber (Fig 5a). Topping the central chamber of Dolmen 3 is a single giant, basalt capstone. The stone, irregular in shape, measures over 4 meters in length, 3.5 meters in width and more than 1.2 meters in thickness, with an estimated weight of over 50 tons (Figs 5 and 6). This is one of the largest stones reported to have been used in the construction of a dolmen in the Levant. On the southeast corner of the central chamber ceiling, the panel of engraved rock art was discovered (Fig 6 & see below). For comparison, at Tell er-Ras in Jordan, the average limestone roof-stone measures 300 x 250 x 50 cm, weighing, therefore, approximately 4 tons, while the average side-stone weighs 1.5 tons. Bahat estimated the weight of the capstone of the dolmen he excavated to be c. 30 tons [20]. Dolmen 3 is, therefore, a unique, monumental, multi-chambered dolmen, with a hierarchical structure: a central chamber roofed by a gigantic engraved capstone and surrounded by a giant tumulus into which at least 4 additional sub-chambers were built. This is the first reported complex “multi-dolmen” in the Levant. It should be noted that the adjacent Dolmen 4 is of the same multi-dolmen type (Fig 3).

## Excavation of Dolmen 3 central chamber

**Fig 7:**
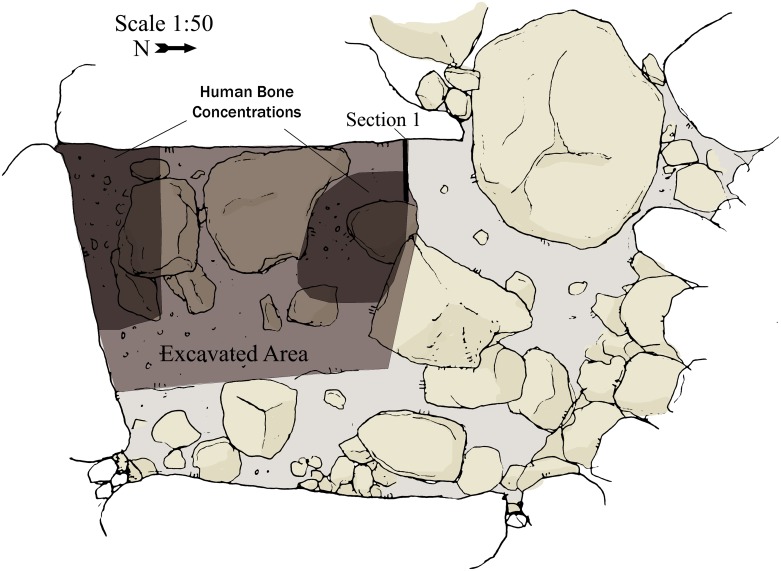
Dolmen 3 central chamber 3a floor plan at the beginning of excavation. Location of excavated area, bone concentration and Section 1 are marked.

Excavation of the central chamber of Dolmen 3 focused on the southwest quarter of the chamber (Fig 7). The objective was to expose the stratigraphic sequence of the accumulation while leaving a significant section of the sediments available for future investigation. Excavation was careful and slow inside the dark, difficult to access chamber. It is now possible to reconstruct the stratigraphic sequence of the dolmen and its implications for the post-depositional accumulation processes, construction, and burial praxis of this megalithic structure.

### Stratigraphy

**Fig 8:**
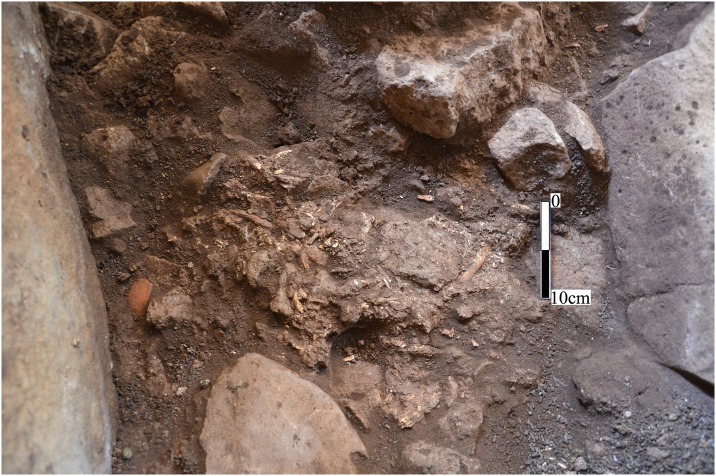
Bones and ceramic remains exposed in central chamber 3a. Scale 10cm.

**Fig 9:**
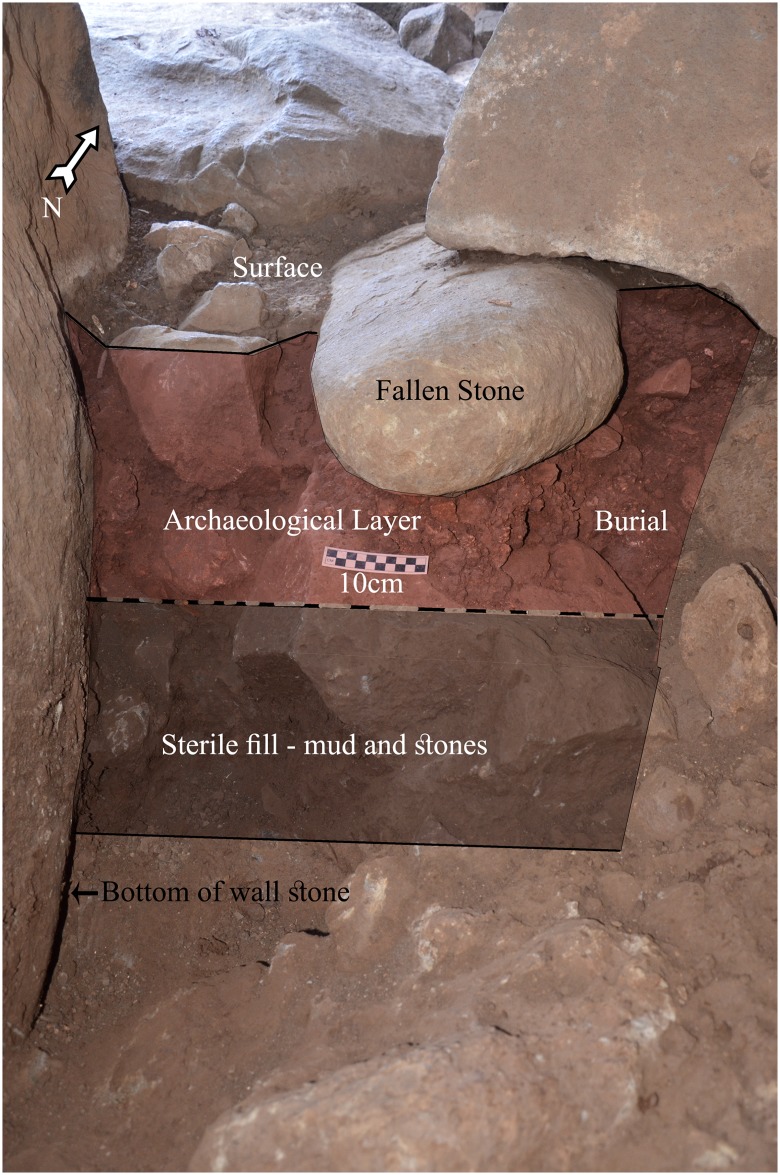
Dolmen 3 central chamber 3a section 1 (for location see Fig 7). Scale 10cm.

The stratigraphic sequence of the dolmen 3a chamber comprises the following layers, from top to bottom (Figs 8 and 9): The pre-excavation surface of the chamber was comprised of large stones and slabs (>50cm), many of which were scattered alongside the chamber walls (Fig 5b). These stones were most likely moved and reorganized during later visits to the chamber. The stones and slabs were either part of the original dolmen floor or, more likely, collapsed inward (e.g. smaller stones used in construction to fill in gaps between large orthostatic stones). In either case, it is impossible to discern the form of the original surface. It should be noted that excavation to date of all other chambers in the Shamir Dolmen Field and in other fields in the Golan always exposed a basalt slab paved floor [25]. Between these stones was loose, dusty soil, containing hyrax dung. The surface soil was only some 5 cm in thickness. In all of the dolmens excavated by Epstein in the Golan [25] there was a stone and earth fill that accumulated over the years and contained Byzantine and later material. Such fill was absent from chamber 3a, the central chamber of Dolmen 3.

Immediately below the surface was a layer of dark soil containing numerous heavily fragmented bones and ceramics. The finds were concentrated in the southwest corner and in the middle of the excavated area (Fig 7). This archaeological horizon is 20 to 30 cm thick. Below the archaeological horizon is a thick (>40cm), archaeologically sterile layer of fine, dark sediment with numerous angular basalt cobbles and pebbles. The bottom of the gigantic, orthostatic side-stones forming the walls of the chamber was exposed at a depth of about 40cm into this layer (Fig 7).

The archaeological horizon of chamber 3a is very close to the surface. Below the archaeological layer is 30 to 40 cm of archaeologically sterile sediment that filled the chamber before the archaeological remains were laid. The following interpretation for this stratigraphy can be suggested: The builders erected the gigantic side-wall slabs, possibly by first digging into the surface of the slope. In the next stage, before placing the giant capstone on top of the chamber walls, the builders filled the chamber with sediment and stones to prevent it from collapsing when placing the 50 ton capstone. Once the capstone was in place, the builders removed a portion of the fill, leaving some 40 cm of sediment without levelling the surface. The human bones and ceramics grave goods were laid directly onto this uneven surface. In addition, sections of the chamber surface were covered by large slabs with only sterile sediments beneath them. It is impossible to say whether the burials were originally covered by stone slabs.

### Human remains

The excavation exposed a series of what appear to be secondary burials, unearthed only a few centimeters below surface. Concentrations of heavily fragmented bones were found in the sediment and between large basalt stones in the southwest corner and in the center of the chamber (Fig 7). Some of the bones were found immediately adjacent to the chamber wall (Fig 8) and some were partially covered by basalt slabs. Each of these bone concentrations contained the remains of more than a single individual. Despite the heavy fragmentation, the preservation of the bone tissue was surprisingly good and it was possible to identify remains from all parts of the skeleton, from skull to leg bones, in each concentration.

The small identified bone fragments include at least three petrous parts of the temporal bone, including that of a child [28]; several deciduous and permanent teeth, of an 8–10 year-old child, a young adult and a 35–45 year-old adult; portions of the axial and appendicular skeleton of both adults and children, including a small pelvic fragment of the greater sciatic notch and hence probably of a female. Altogether, the Dolmen 3 burial includes at least three individuals: an adult male and female, and a young child. The distribution of bone fragments from different individuals indicates a multi-burial. As noted above, given the likelihood of a later disturbance of chamber 3a, reconstruction of the original context is difficult. This disturbance, together with the finding of bones from at least three individuals, indicates a complex burial praxis possibly involving the pushing aside of previously buried bones during subsequent burials after an unknown passage of time.

The burial praxis of Dolmen 3 is similar to that described by Epstein [25] for many of the Golan dolmens. Human bones, including fragments of long bones, skull bones, and teeth, were found in many of the Golan dolmens. However, the poor preservation of the bones (and of the ceramic sherds) due to moist conditions inside the chambers hampered further research. In many cases bones were pushed aside to make room for later burials indicating, as Epstein noted, that the burials were secondary. Epstein suggested that perhaps not all skeletal bones were reburied in the dolmens. Epstein assigned the post IB (MBI in Epstein’s terminology) finds from many of the Golan dolmens to reuse of the dolmens for burial in later periods. Reuse may also explain the similar finds here from Dolmen chamber 3a –secondary burial of a few individuals with IB ceramic grave offerings that were possibly disturbed by later use of the chamber. The Shamir dolmen chamber 3a burials are unique in that the good preservation enabled us to define the minimum number and age of the individuals in the bone assemblage. It is currently not possible to conclude whether the burials were found as placed during the original use of the dolmen or as disturbed by later burials, suggested by the presence of later period beads (see below).

### The chronology of Dolmen 3

Since their first discovery over 100 years ago, the chronology of the Levantine dolmens has been in debate. These dolmens are large structures, dominating the landscape, and many were either robbed or reused over the majority of their existence. The combination of heavily disturbed depositional history and poor preservation conditions resulted in the absence of reliable datable material from most dolmens excavated in the region [25]. To date, not a single radiometric date was obtained from a Levantine dolmen. An effort to obtain a ^14^C chronology from the bones excavated from Dolmen 3 is ongoing, but the poor chemical preservation of the bones is challenging the dating process. Levantine dolmens have been assigned chronologically to the Pre-Pottery Neolithic (based on the absence of ceramics, e.g. [29]), the Chalcolithic, all stages of the Bronze Age and to later periods (for overview see [3,8]). Researchers have achieved substantial advances in the study of the Levantine dolmens in recent decades, primarily for the dolmens located in the east escarpments of the Jordan Rift Valley south of the Golan. They have surveyed numerous dolmen fields, excavated many structures, and determined their chronology (e.g. [30–33]). All evidence suggests that east of the Jordan Rift Valley, south of the Yarmuk River, burial in dolmens was a common practice during the Early Bronze Age I (4^th^ millennium BC). These new discoveries support the suggestion that the dolmens of the Golan (north of the Yarmuk River) and Galilee should be distinguished from the dolmens east of the Southern Jordan Rift Valley. The Golan dolmens differ from the southern dolmens in the type of rock used for their construction (basalt vs limestone and sandstone), in size (they are typically much larger), in their design (typically passage tombs covered by a constructed tumulus), building technology (unworked stones), and in their chronology. It seems that the Golan and Galilee dolmens find their cultural roots in the north, in the megalithic traditions of Syria and Anatolia. In all of the many dolmens excavated and surveyed in the Golan and its escarpments, the earliest material unearthed clearly belongs to the Intermediate Bronze Age [24,25,34].

**Fig 10:**
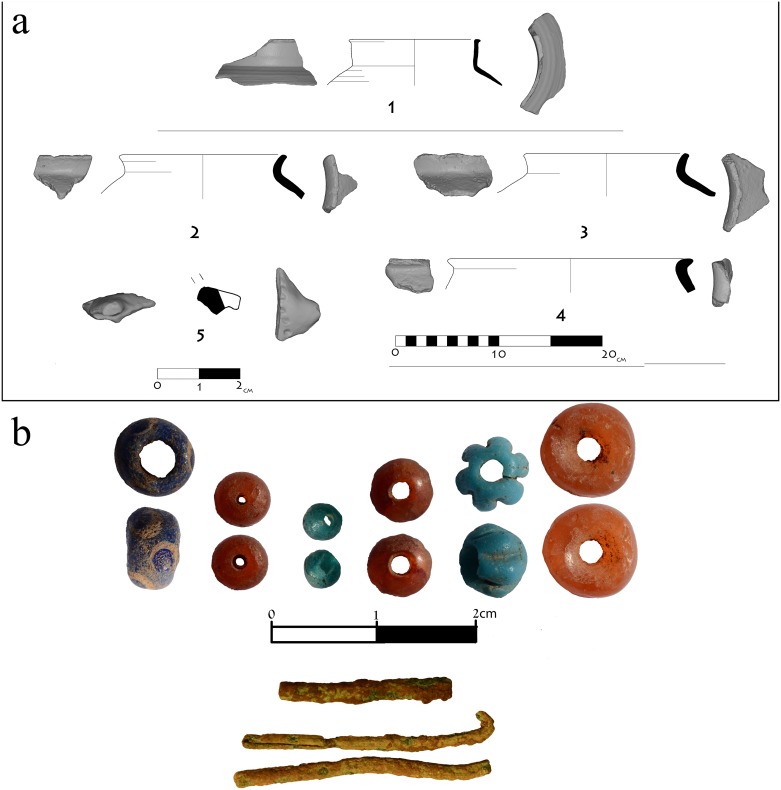
Finds from Dolmen 3 excavation. a. Ceramic remains. b. Beads.

All finds to date from the Shamir Dolmen Field support IB chronology [24] and Dolmen 3 is no exception. Similar to all other dolmens excavated in the Levant, dating is based upon the chamber finds, particularly on ceramic evidence. In Dolmen 3, numerous ceramic sherds were uncovered in the immediate context of the human bones (Fig 8). These sherds are probably the remains of grave goods offered at burial. All of the in situ sherds found in Dolmen 3 were assigned to the IB (Fig 10a) as they fall well within the ‘north family’ of IB ceramics (see overview and references in [22,24,25]). The ceramic evidence is the primary chronological marker for the building of Dolmen 3. A single fragment of a Roman cooking pot (Fig 10a -1) was collected from the surface of chamber 3a. It indicates a later visit to the Shamir Dolmen Field, but it should be noted that this fragment is minimal evidence in comparison to finds from most other dolmens excavated in the Golan [25]. Dating of the chamber 3a burials is somewhat less secure due to the presence of colorful beads of different sizes and shapes collected during the sieving of the sediments excavated, as well as small metal pieces (Fig 10b), possibly the remains of a fibula pin. The metal pieces can easily be attributed to the IB, as they are similar to finds unearthed from other dolmens in the Golan as well as from the Shamir Burial Cave [24,25]. However, some of the beads can only be assigned to later periods according to the region’s bead chrono-typology [35]. We suggest viewing the beads as an intrusion of material into the dolmen, but it may be that the burials, or at least some of the burials, should be assigned to later reuse of the dolmen. Nevertheless, the overwhelming evidence suggests that the actual construction of Dolmen 3 is well-dated to the IB.

### The rock art

**Fig 11:**
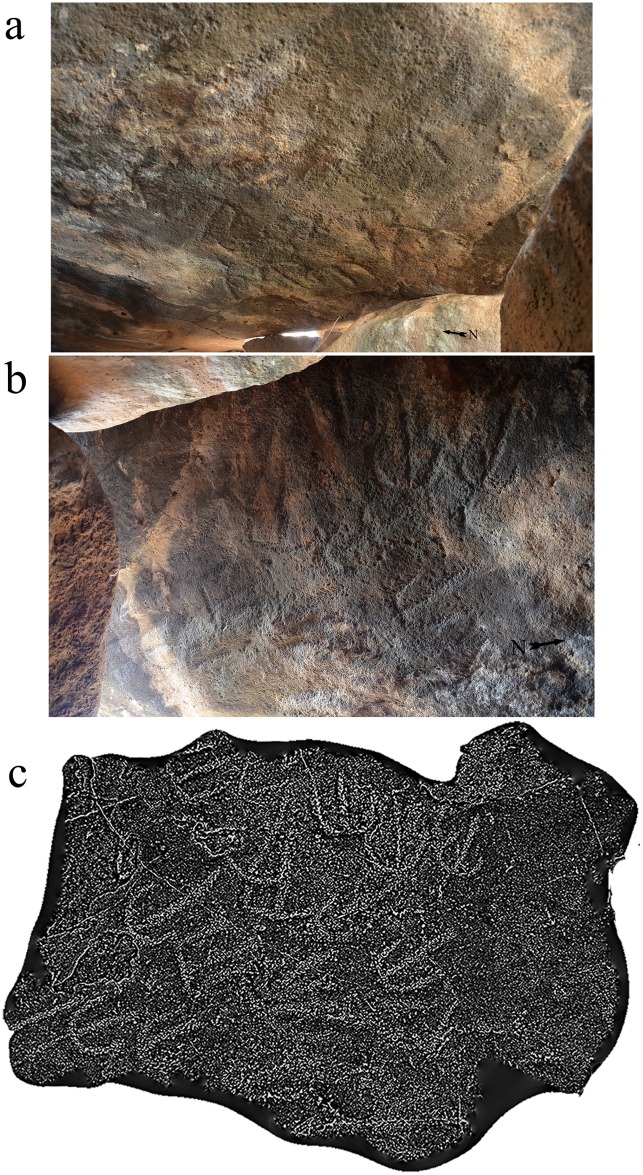
The rock art panel engraved on the ceiling of Dolmen 3 central chamber 3a. a. General view looking toward the northeast. b. General view from below. c. 3D model of the panel.

**Fig 12:**
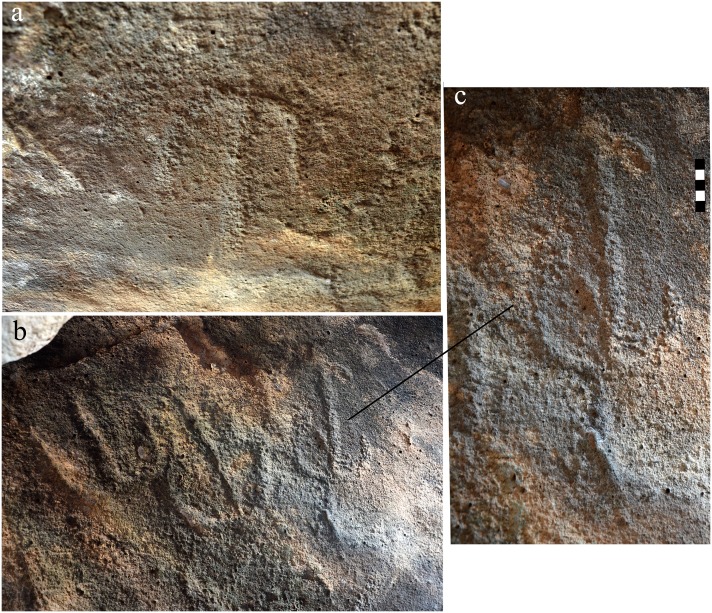
Rock art panel detail.

The unexpected discovery of an engraved panel on the Dolmen 3 central chamber ceiling precipitated this study. The ceiling panel, located at the southeast quarter of the chamber ceiling (Figs 5 and 6), includes fourteen clearly identified schematic, engraved elements (Fig 11). The forms represent variations on a single motif, comprising a vertical line with a downturned arc attached to its upper part (Fig 12). The length of the central line differs between elements as does the curvature of the arc. The average size of the elements is about 25 cm. The alignment of each individual element is slightly different, creating an arced path of movement across the chamber ceiling, from northeast to southwest (Fig 11). The forms were made by pecking into the face of the basalt rock. The inner surface of the engraved lines is relatively uniform and could have been made by chisel or hammer/axe either of metal (bronze) or stone such as flint.

While most of the forms are discernable to the naked eye, some are obscured. The unevenness of the basalt surface, which is weathered around the panel margins, the darkness inside the chamber, and the difficult positioning of the panel all hamper clear identification of the shapes. Even for the more visible forms, it is difficult to determine their exact outline. In order to obtain precise and detailed documentation, 3D-scanning by structured-light technology was used to reveal the subtle secrets of the engraved panel. Several scans run from various directions were registered together into one 3D model. The model was transformed into a triangular mesh, in which every triangle represents the smallest unit of a geometrical plane, and the collection of triangles is an accurate visualization of the geometry of the panel. The model was first rendered in gray and (Fig 11c) then “valleys” of the striations [36] were enhanced on the panel surface. This revealed and sharpened fourteen complete engraved forms and two additional shapes that are indeterminable (Fig 11c). The 3D model was then processed by *Artifact3-D software* [37], which revealed that a different technique was applied to create the straight and arced line of each form. The center, straight lines most likely were created by applying a continuous, back and forth engraving motion. The width of the line was determined by the width of the tool used. This technique would be cumbersome to produce an arced line and, indeed, the 3D model suggests that pecking was the preferred technique for the arced lines. The application of two different techniques for the different line shapes is evidenced by the difference in depth between the straight and curved line of each form.

Similar to megalithic rock art worldwide, the Shamir line and arc motif is abstract in nature (e.g. [15]). While arcs are commonly depicted in this context (see [22]; Fig 11), no exact parallel exists for the Shamir vertical line and arc composition. Two dolmen-like structures with rock art were found in Yemen. Dated to the late 4^th^-3^rd^ millennium BC, the engravings are of geometric designs, including denticulate patterns, crosshatched diagonal rectangles, and rings. They are engraved on the inner faces of the upright stones, and on both the internal and external faces of the capstones [10,11]. The designs form repetitive patterns with no direction or variation.

The meaning of rock art and its function within the megalithic context remains ambiguous (e.g. [38,39]) and the engravings on the Shamir capstone are equally challenging to interpret. However, given the burial context and the placement of the engravings above the human remains, a possible interpretation is that they are schematic human forms or symbolic representations of the soul of the deceased. From this depiction, one may postulate the meaning of the panel as representing, or relating to, the journey that awaits the deceased.

Dating rock art is difficult. It is even more complicated when the art is out of context as in the case of the vast majority of rock art found in the Southern Levant deserts [40,41]. The Shamir panel is unique as it is found in an archaeological context, next to a burial dated to the IB. The engravings can be associated with the actual building of the dolmen and the burial in it for the following reasons: a) the engraved forms are similar in patination to the untouched rock face; b) the hidden location of the panel inside the blocked chamber refutes the possibility that it is a territorial or other land marker or declarative art; c) the location of the panel above the burial supports a symbolic meaning for the engravings; and d) the motif has no known parallel in Levantine rock art and cannot be attributed to a later period. These findings support the in situ nature of the rock art panel, placing it in the context of the construction of the dolmen in which it was engraved. As such, it is one of the rare cases in which early Levantine rock art can be dated and explored, in this instance, in the context of a complex megalithic burial site.

## Discussion

Until recently, the Intermediate Bronze Age of the Levant was understood by researchers as the “Dark Ages” between two urban periods. The collapse of the Early Bronze cities, the near absence of settlements in the archaeological record, together with no reported monumental buildings or any other indicators of a central regime, led to the definition of the socio-economic structure of the IB as “small-scaled mixed agro-pastoralism” [1,4]. The findings from the Shamir Dolmen Field challenge this view and suggest that, at least in the Hula Valley Basin and the Northern Golan Heights, a governmental body existed that had the ability to recruit the labor and organization needed for the stonemasonry of monumental architecture. The complexity of the social organization is further supported by a hierarchical burial complex and the presence of symbolic rock art in the largest dolmen of the vast Shamir Dolmen Field.

In recent decades, study of the dolmens on the eastern escarpments of the Jordan Rift Valley south of the Golan has advanced our knowledge of these megaliths. Based upon the ceramics and other evidence, it is now possible to date this megalithic burial tradition to the EB I societies of the region (e.g. [30,33,42,43] and see overview in [3]). In contrast, while researchers have studied dolmens in the Golan and Galilee for over 150 years, the number of modern excavations performed is limited and, in many cases, restricted to a small number of dolmens as part of a salvage excavation [3,8,9,25]. The majority of excavated dolmens were found to be devoid of artifacts, most likely a combination of poor preservation conditions and grave robbing in ancient times. This lack of finds prohibited establishing a chronology and defining the usage of the structures. Currently, the construction of all dolmens in the Golan have been dated to the IB [8,25,27]. The in situ finds from the excavation of Shamir Dolmen 3 are in full agreement with this chronological framework.

Shamir Dolmen 3 stands out as a monumental structure within the IB landscape of the Hula Basin. Hundreds of tons of basalt stones were moved to build the mega-structure that was then covered by a giant basalt capstone weighing some 50 tons. Dolmen 3 is a hierarchical structure with a central chamber housing multi-burials and a ceiling decorated by rock art, surrounded by smaller sub-chambers within the same tumulus complex. Such hierarchy suggests a complex governmental system, possibly composed of stratified social classes.

Recently, Fraser [3], based on a calculation by Arnold [44], criticized the view that the amount of labor invested in dolmen construction is an indicator of a complex governmental system (tombs for elite; e.g. [27]). However, the calculations were applied to the much smaller dolmens of Jordan south of the Golan. The Dolmen 3 fifty ton capstone and the hundreds of additional tons of basalt used to construct the tumulus indicate a much larger scale of labor and time. A complex governmental system was needed to recruit laborers for building such a monumental structure and for supplying their needs during the operation. It also needed to possess the architectural knowledge and dexterity for the complex stonemasonry involved. Shamir Dolmen 3 is surrounded by a large concentration of dolmens that, while smaller in size, are also huge structures that required social organization, knowledge, and labor for their building. Moreover, while rock art has been documented in European dolmens and megalithic structures, no rock art has previously been reported from dolmens in the Southern Levant [3]. It is notable that the only such rock art reported in the region to date is found in the largest and most complex structure of the Shamir Dolmen Field. This finding is an additional indicator for the complex and advanced praxis associated with these dolmens.

The Shamir Dolmen Field comprises hundreds of dolmens, most of which were built using uniform architecture: a central rectangular chamber surrounded by a well-built circular tumulus, covered by a giant capstone visible from afar. This uniformity suggests a cultural pattern that was practiced over a significant period of time. The Shamir Dolmen Field is not the only monumental megalithic structure of the Golan. Thirty km to the southeast is the famous Rujm el Hiri monument [45]. The chronology and function of this megalithic circle, with a diameter of 160 meters, are still under debate [9]. Similar to the Shamir Dolmen Field, Rujm el Hiri is a monumental structure, surrounded by additional megalithic structures overlooking it from the surrounding hills, with no significant settlement system in the vicinity. It is evident that the Rujm el Hiri megalithic “tradition” is similar, yet not identical, to the Shamir dolmen megalithic tradition. This similarity may indicate neighboring nomadic socio-political systems, sharing comparable megalithic burial praxes as their governmental or ceremonial symbols.

## Conclusion

The Shamir Dolmen Field is evidence of a hierarchical, complex society with well-defined burial customs and monumental architectural design, requiring a great deal of human labor and effort. Complex burial customs, a hierarchical burial system, and symbolic rock art all suggest a much more complex socio-economic system in the Hula Valley Basin than thought possible in the Levantine Intermediate Bronze.
